# COGNITION AND PAIN ARE ASSOCIATED WITH DISCREPANCIES IN ACTIGRAPHY AND SELF-REPORTED SLEEP MEASURES IN OLDER ADULTS

**DOI:** 10.1093/geroni/igae098.0520

**Published:** 2024-12-31

**Authors:** Russell Calderon, Sofia Liu, Miranda McPhillips, Michelle Liu, Yifan Liu, Junxin Li

**Affiliations:** Johns Hopkins University, Lehigh Acres, Florida, United States; Johns Hopkins University School of Nursing, Baltimore, Maryland, United States; Villanova University, Villanova, Pennsylvania, United States; Johns Hopkins University, Baltimore, Maryland, United States; Johns Hopkins University, Baltimore, Maryland, United States; Johns Hopkins School of Nursing, Baltimore, Maryland, United States

## Abstract

Sleep is a key determinant of health in older adults and is assessed using accelerometer-based (Actigraphy) and self-reported methods. This study aims to describe and identify factors associated with potential discrepancies between Actigraphy and self-reported sleep parameters (“Actigraphy/self-reported discrepancies”) in 125 community-dwelling older adults without dementia [Montreal Cognitive Assessment (MoCA) ≥17] using data from a randomized clinical trial (NCT03959202). Participants were 70.4±6.3 years old, 79.2% females, 21.6% with mild cognitive impairment. Actigraphy and corresponding self-reported sleep measures, including time in bed (TIB), sleep onset latency (SOL), total sleep time (TST), and sleep efficiency (SE) were collected using Actiwatch 2 for 7-10 days, sleep diaries, and Pittsburgh Sleep Quality Index (PSQI). Relative to Actigraphy measures, 11.2%, 38.4%, 52.0%, 35.2%, and 36.0%, of participants reported longer TIB(diary) (>15 minutes), TIB(PSQI) (>15 minutes), SOL(PSQI) (>5 minutes), TST(PSQI) (>15 minutes), and better SE(PSQI) (>5%) respectively, whereas 38.4%, 52.0%, 12.0%, 54.4%, and 28.0% of participants reported shorter TIB(diary), TIB(PSQI), SOL(PSQI), TST(PSQI), and worse SE(PSQI) by the same magnitudes. Multiple linear regression analyses adjusted for demographics suggest higher MoCA was associated with reduced Actigraphy/self-reported discrepancies (TIB(Actigraphy-diary):β=-13.6, p=0.037; TIB(Actigraphy-PSQI):β=-11.2, p< 0.01; SOL(Actigraphy-PSQI):β=-3.3, p=0.019; TST(Actigraphy-PSQI):β=-7.4, p=0.01; SE(Actigraphy-PSQI):β=-1.5, p=0.013). Higher pain behavior expression (TIB(Actigraphy-PSQI):β=7.3, p=0.011; SOL(Actigraphy-PSQI):β=2.7, p=0.014) and pain interference (TIB(Actigraphy-diary):β=8.8, p< 0.01) were associated with greater Actigraphy/self-reported discrepancies. In summary, cognitive function and pain were associated with Actigraphy/self-reported discrepancies among older adults without dementia. Given that cognition and pain may interfere with self-perceptions of sleep quality, sleep must be carefully assessed and interpreted in this population.

